# Growth Curve of Disability of Older Adults Over a 12-Year Period: Can It Be Modified by Age or Engaging in Activities?

**DOI:** 10.1093/geroni/igaa057.1295

**Published:** 2020-12-16

**Authors:** Rongjun Sun

**Affiliations:** Cleveland State University, Cleveland, Ohio, United States

## Abstract

Adopting a growth curve model, this study aims to fit a growth trajectory of disability of older adults over a 12-year period, and to investigate whether such a trajectory is modified by initial age and level of engagement in activities. The data are from the Chinese Longitudinal Healthy Longevity Survey, which includes 16,064 individuals aged 60 or above in the first wave in 2002 who were followed-up in four more waves until 2014. Disability in this study is measured by having any difficulty in performing six activities of daily living. Activities include physical exercise and eight leisure activities. To rigorously test the causal effect of engaging in activities on disability, we adopted a time-lagged growth curve model. In addition, disability status in the initial wave was controlled at baseline and an array of health status measures, such as physical functioning and cognitive performance, were included as time-varying covariates. We introduced a random effect to control for unobserved heterogeneity between individuals. The results show a quadratic curve of disability over time with an accelerating pace in later waves. While initial age shows a moderate modifying effect, engaging in leisure activities substantially modified the trajectory: The probability of being disabled increased from 6.7% to 45.8% between the first and fourth follow-up for those inactive individuals. For those active older adults, it only increased from 3.1% to 18.0%. This study demonstrates that engaging in leisure activities can significantly reshape the trajectory of developing disability among older adults.

